# Elastostatics of Bernoulli–Euler Beams Resting on Displacement-Driven Nonlocal Foundation

**DOI:** 10.3390/nano11030573

**Published:** 2021-02-25

**Authors:** Marzia Sara Vaccaro, Francesco Paolo Pinnola, Francesco Marotti de Sciarra, Raffaele Barretta

**Affiliations:** Department of Structures for Engineering and Architecture, University of Naples Federico II, Via Claudio 21, 80125 Naples, Italy; marziasara.vaccaro@unina.it (M.S.V.); francescopaolo.pinnola@unina.it (F.P.P.); marotti@unina.it (F.M.d.S.)

**Keywords:** Wieghardt foundation, Bernoulli–Euler beams, nonlocal effects, integral nonlocal model

## Abstract

The simplest elasticity model of the foundation underlying a slender beam under flexure was conceived by Winkler, requiring local proportionality between soil reactions and beam deflection. Such an approach leads to well-posed elastostatic and elastodynamic problems, but as highlighted by Wieghardt, it provides elastic responses that are not technically significant for a wide variety of engineering applications. Thus, Winkler’s model was replaced by Wieghardt himself by assuming that the beam deflection is the convolution integral between soil reaction field and an averaging kernel. Due to conflict between constitutive and kinematic compatibility requirements, the corresponding elastic problem of an inflected beam resting on a Wieghardt foundation is ill-posed. Modifications of the original Wieghardt model were proposed by introducing fictitious boundary concentrated forces of constitutive type, which are physically questionable, being significantly influenced on prescribed kinematic boundary conditions. Inherent difficulties and issues are overcome in the present research using a displacement-driven nonlocal integral strategy obtained by swapping the input and output fields involved in Wieghardt’s original formulation. That is, nonlocal soil reaction fields are the output of integral convolutions of beam deflection fields with an averaging kernel. Equipping the displacement-driven nonlocal integral law with the bi-exponential averaging kernel, an equivalent nonlocal differential problem, supplemented with non-standard constitutive boundary conditions involving nonlocal soil reactions, is established. As a key implication, the integrodifferential equations governing the elastostatic problem of an inflected elastic slender beam resting on a displacement-driven nonlocal integral foundation are replaced with much simpler differential equations supplemented with kinematic, static, and new constitutive boundary conditions. The proposed nonlocal approach is illustrated by examining and analytically solving exemplar problems of structural engineering. Benchmark solutions for numerical analyses are also detected.

## 1. Introduction

Structural models of beams on an elastic foundation have been widely exploited by the scientific community to describe engineering problems with numerous applications in geotechnics, road, railroad, marine engineering, and biomechanics; see e.g., [1].

The problem of a beam subjected to transverse distributed loading proportional to its deflection was considered by E. Winkler in the framework of the local theory of elasticity [2]. It was then considered to model railway tracks on continuous linear elastic foundations by H. Zimmermann in his handbook on railway constructions [3]. Winkler and Zimmermann’s theory quickly had followers due to its simplicity and easy mathematical treatment since the soil was modeled in terms of one parameter as a continuous bed of independent linear elastic one-dimensional springs with uniform stiffness.

However, E. Wieghardt [4] remarked that, in spite of its intuitive nature, Winkler’s model was not physically fully reliable since it predicts sharp discontinuities in the beam-soil profile at beam ends that are not actually present in real phenomena. However, Winkler’s approach can be effectively exploited to address other engineering applicative problems, such as floating bridges and ice sheets [5,6,7,8], cemented lap joints [9,10], timber facings [11], grillage structures [12,13], shrink-fit problems [14], cracks [15,16], and cracked plates [17,18].

In 1922, Wieghardt proposed a model in which the deflection at each point of the surface of the foundation depends on the response of the entire contact region beam-soil through an integral of soil reactions weighted by a suitable kernel function. The mathematical model thus depends on a stiffness parameter and on an additional parameter entering the averaging kernel. Later on, this problem was tackled by W. Prager [19] and P. Neményi [20] for two-dimensional foundations.

Subsequently, in the framework of the local theory of elasticity, two different soil models characterized by two material parameters were introduced. The former was proposed by M. Filonenko-Borodich [21] assuming that a membrane under tension is interposed between beam and Winkler-type elastic springs. The latter was provided by P. Pasternak [22] by considering a shear interaction among elastic springs modelling the soil. A discussion on formulations of beam-soil and plate-soil interactions can be found in the review paper by Wang et al. [23].

### Winkler and Wieghardt Foundations

Let us consider a straight beam, with a cross section Ω, of length *L* laying on an elastic foundation. The *x*-coordinate is taken along the beam length, the *y*-coordinate is along the thickness (height), and the *z*-coordinate is along the beam width originating at the cross-sectional elastic centre *C*. The pair $\left\{y,z\right\}$ collects principal axes of geometric inertia of the cross section Ω.

The classical Winkler theory of a continuous medium supporting a beam (see e.g., [2,3]) considers the elastic foundation composed of an infinite sequence of linear elastic springs, and at each point, the foundation reaction per unit length is directly proportional to the deflection of the foundation. The elastic foundation is characterized by a positive parameter β representing the pressure to be orthogonally applied to the surface to obtain a unit vertical displacement of the surface of the foundation. The modulus β is a volumetric density of force, and denoting by *b* the width of the beam cross section in contact with the surface of the elastic soil, the related stiffness is given by k=βb. Hence, the relationship between reaction per unit length r(x) applied to the foundation surface and transverse displacement v(x) is

(1) r(x)=kv(x).

We assume that the beam remains in contact with the foundation surface so that the transverse displacement *v* of the beam coincides with the transverse displacement of the surface of the foundation. According to the classical (local) Winkler elastic model provided by Equation (1), the reaction at a point *x* is proportional to the displacement at the same point. Hence, the mechanical model of the Winkler elastic foundation is illustrated by linear springs unconnected with each other.

The refinement originally proposed by Wieghardt [4], afterwards analyzed in [24,25,26,27], introduced the assumption that the transverse displacement *v* at a point of the foundation surface depends on reactions *r* on other points of the foundation in a nonuniform way in terms of the following Reaction-Driven (RD) integral convolution law

(2) $$v\left(x,L_{c}\right)=\int_{0}^{L}\phi \left(x-t,L_{c}\right)\frac{r\left(t\right)}{k}dt.$$

The smoothing kernel ϕ depends on the characteristic length of Eringen nonlocal elasticity $L_{c}=\lambda L$, with λ>0 being a non-dimensional nonlocal parameter, and is given by the bi-exponential averaging function; see, e.g., [28,29]:

(3) $$\phi \left(x,L_{c}\right)=\frac{1}{2L_{c}}\exp\left(-\frac{\left|x\right|}{L_{c}}\right).$$

The maximum value of the bi-exponential function is obtained at x=0 for any $L_{c}$ and decaying to zero at suitable distances. Thus, the Wieghardt model is of nonlocal nature and this aspect makes it different from all the others, where basically the response at a point depends on the displacement at that point.

## 2. Research Significance, Motivation, and Outline

It has to be pointed out that it is not possible to solve, in general, the nonlocal elastostatic problem of a beam on a Wieghardt elastic foundation modelled by Equation (2), as highlighted by Wieghardt himself and discussed by T. Van Langendonck [24], A. Sollazzo [25], A. Ylinen, and M. Mikkola [26].

In fact, the RD (Reaction-Driven) formulation of the Wieghardt elastic foundation Equation (2) can be equivalently rewritten in the following differential form:

(4) $$\frac{1}{L_{c}^{2}}v\left(x\right)-\partial_{x}^{2}v\left(x\right)=\frac{1}{kL_{c}^{2}}r\left(x\right),$$

with $x\in \left[0,L\right]$, subject to two RD Foundation Boundary Conditions (RDFBCs):

(5) $$\left\{\begin{array}{c} {\left.\partial_{x}v\left(x\right)\right|}_{x=0}=\frac{1}{L_{c}}v\left(0\right)\\ {\left.\partial_{x}v\left(x\right)\right|}_{x=L}=-\frac{1}{L_{c}}v\left(L\right). \end{array}\right.$$

Proof of the result above is analogous to the one in [29] regarding Eringen’s internal elasticity theory.

It is then apparent that ill-posedness of a Bernoulli–Euler beam on an RD model of Wieghardt elastic foundation is related to the incompatibility between kinematic boundary conditions and RDFBCs. In fact, Equation (5) forces the rotations $\varphi \left(x\right)=\partial_{x}v\left(x\right)$ of the beam end cross-sections to coincide to corresponding transverse displacements divided by the nonlocal parameter $\pm L_{c}$. Such requirements are not met by most of kinematic boundary conditions of beams involved in technical applications.

In order to by-pass the ill-posedness of the elastostatic problem of a beam on Wieghardt elastic foundation, two fictitious reactive forces exerted by the soil were introduced at the beam end points [25]. Accordingly, the Modified Reaction-Driven (MRD) nonlocal model of Wieghardt elastic foundation was introduced by defining the transverse displacement *v* of the surface of the elastic foundation in terms of the reaction *r* and of two fictitious forces $A_{1}$ and $A_{2}$ in the following form:

(6) $$v\left(x\right)=\int_{0}^{L}\phi \left(x-t,L_{c}\right)\frac{r\left(t\right)}{k}dt+\frac{A_{1}}{2L_{c}k}\exp\left(-\frac{x}{L_{c}}\right)+\frac{A_{2}}{2L_{c}k}\exp\left(\frac{x-L}{L_{c}}\right).$$

The elastic equilibrium problem of a beam on the modified Wieghardt elastic foundation is reported for completeness in Appendix A. It is then apparent that the two fictitious forces were added in order to match the number of unknowns of the problem with the six boundary conditions of the elastostatic nonlocal differential problem; see also [30].

The motivation of the present paper consists in formulating a well-posed nonlocal integral model of elastic foundation such that no fictitious forces are postulated at the end points of Bernoulli–Euler beams in order to solve the relevant structural problem. Specifically, the nonlocal model of elastic foundation is cast in the framework of Eringen theory [31,32,33] requiring that reaction fields are outputs of convolutions between displacement fields of the elastic foundation and a suitable averaging kernel. Such an approach of external elasticity is thus named the displacement-driven nonlocal model.

It is worth recalling that, as acknowledged by the scientific community, Eringen’s strain-driven nonlocal model of internal elasticity is inapplicable to structural problems of applicative interest due to incompatibility between equilibrium and constitutive requirements [34,35]. On the contrary, no conflict is present if the elastostatic problem of a Bernoulli–Euler beam resting on elastic foundation is formulated by considering the displacement-driven nonlocal model of external elasticity.

However, the corresponding equations of elastic equilibrium are described by complicated integrodifferential laws [36], the solution of which requires utilization of advanced computational procedures. A skillful Finite Element Method (FEM) strategy was indeed conceived in [37] to solve nonlocal dynamical problems of viscoelastic structures.

In this paper, the integrodifferential elastic problem of a beam resting on a displacement-driven nonlocal foundation is shown to be equivalent to a much simpler differential problem that can be analytically solved without any additional complication with respect to classical Winkler equations. The key idea consists in proving that the convolution integral describing the nonlocal model of an elastic foundation is equivalent to an ordinary second-order differential equation equipped with nonstandard constitutive boundary conditions. The new approach is exploited to investigate the bending behavior of Bernoulli–Eulerelastic beams on displacement-driven nonlocal foundation for a variety of boundary kinematic constraints of technical interest. The effects of nonlocal parameters and the stiffness coefficient of the Winkler elastic soil on structural transverse displacements, reactions, bending, and shear forces are analytically evaluated and compared with outcomes in literature.

## 3. Bernoulli–Euler Beams on Elastic Foundation

In a Bernoulli–Euler beam, applied loads and geometry are such that the displacements $\left(s_{x},s_{y},s_{z}\right)$ along the coordinates $\left(x,y,z\right)$ are functions of the *x*- and *y*-coordinates and are given by

(7) $$s_{x}\left(x,y\right)=-\partial_{x}v\left(x\right)y,\;s_{y}\left(x,y\right)=v\left(x\right),\;s_{z}\left(x,y\right)=0,$$

where *v* is the transverse displacement of the cross section and the symbol $\partial_{x}\left(•\right)$ denotes the derivative of the function • along the nanobeam axis *x*.

The rotation φ of the beam cross section is $\varphi \left(x\right)=\partial_{x}v\left(x\right)$ such that the nonvanishing kinematically compatible deformation is given by the axial strain

(8) $$\varepsilon_{x}\left(x,y\right)=-\partial_{x}^{2}v\left(x\right)y=-\chi \left(x\right)y,$$

where $\chi \left(x\right)=\partial_{x}^{2}v\left(x\right)$ is the kinematically compatible bending curvature of the beam. In the absence of thermal distortions, the kinematically compatible flexural curvature χ coincides with the elastic bending curvature.

The stress resultant moment *M* is

(9) $$M=-\int_{\Omega }\sigma ydA=I_{E}\chi \left(x\right)$$

with $I_{E}$ beingthe second moment of elastic area about the *z* axis of the distribution of Young’s moduli E(y):

(10) $$I_{E}=\int_{\Omega }E\left(y\right)y^{2}dA.$$

The differential equilibrium equation of a beam subject to a distributed transverse load $q_{y}\left(x\right)$ per unit length in the interval [0,L] is given by $\partial_{x}^{2}M\left(x\right)=q_{y}\left(x\right)-r\left(x\right)$ in $\left[0,L\right]$ with the boundary conditions $T\left(x\right)=-\partial_{x}M\left(x\right)=F$, M(x)=M at the beam end point x=L and T(x)=−F, M(x)=−M at x=0 with *T* shear force and (F,M) transverse force and couple, respectively.

Using the differential condition of equilibrium, the definition of bending curvature χ, and Equation (9), we obtain the elastic equilibrium differential equation of the beam on an elastic foundation in the following form:

(11) $$I_{E}\partial_{x}^{4}v\left(x\right)=q_{y}\left(x\right)-r\left(x\right).$$

## 4. Elastic Equilibrium Problem of a Beam on Displacement-Driven Nonlocal Foundation

**Figure 1:**
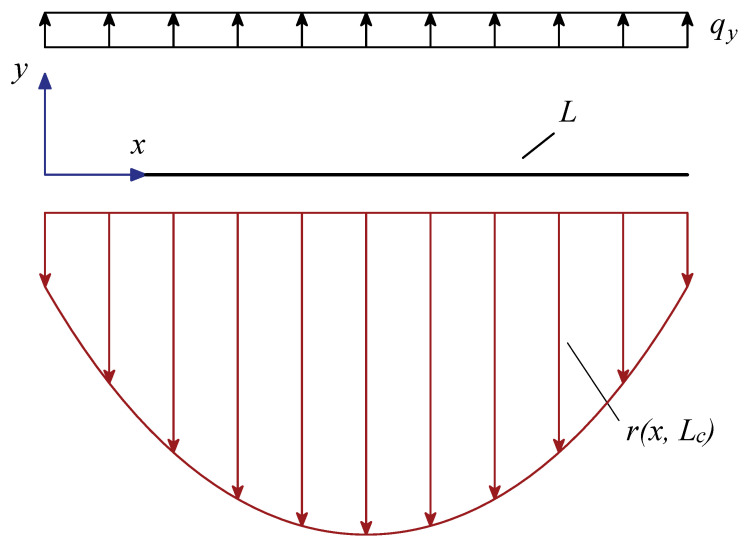
Geometric sketch of a free-beam on displacement-driven nonlocal foundation.

A well-posed nonlocal model of a beam lying on elastic foundation (see Figure 1) is presented below.

Let us assume that reactions *r* are linked to the transverse displacement *v* of the surface of the foundation in correspondence to the beam interval $\left[0,L\right]$ by a Displacement-Driven (DD) convolution integral in the following form:

(12) $$r\left(x,L_{c}\right)=\int_{0}^{L}\phi \left(x-t,L_{c}\right)kv\left(t\right)dt.$$

For simplicity, in the sequel, explicit dependence of *r* on the characteristic length $L_{c}$ is dropped.

The bi-exponential kernel $\phi \left(x,L_{c}\right)$ Equation (3) fulfils normalization, symmetry, and limit impulsivity conditions.

The symmetry condition $\phi \left(x,L_{c}\right)=\phi \left(-x,L_{c}\right)$ of the function ϕ expresses the mechanical assumption that symmetrically placed points of the foundation with respect to the considered point *x* have the same influence on the reactions *r* at *x*. Moreover, the characteristic parameter $L_{c}$ is a measure of how rapidly the influence of the displacement *v* at a point *t* decreases with the distance from the considered point *x*. Denoting by δ(x) the Dirac unit impulse at point *x* inside the structural interval, the impulsivity condition

(13) $$\lim_{L_{c}\to 0^{+}}\phi \left(x,L_{c}\right)=\delta \left(x\right)$$

ensures that Equation (12) yields $r\left(x\right)=kv\left(x\right)$, for $L_{c}\to 0^{+}$, so that the classical Winkler model of an elastic foundation, see Equation (1), is recovered in ]0,L[.

The elastostatic structural problem of a beam resting on nonlocal DD elastic foundation can be formulated by considering the beam elastic equilibrium Equation (11), with classical kinematic and static boundary conditions, and DD convolution integral of the nonlocal elastic foundation Equation (12) as reported in Box 1.

Box 1 **Elastostatic structural problem of a beam on nonlocal displacement-driven DD elastic foundation.**

(14a) $$\begin{array}{cc} \; & I_{E}\partial_{x}^{4}v\left(x\right)=q_{y}\left(x\right)-r\left(x\right)\;\mathrm{Beam}\;\mathrm{elastic}\;\mathrm{equilibrium}\\ \; & \end{array}$$

(14b) $$\begin{array}{cc} \; & \left\{v\left(x\right),\varphi \left(x\right),\right.{\left.M\left(x\right),T\left(x\right)\right\}}_{x=\left\{0,L\right\}}\;\mathrm{Kinematic}\;\mathrm{and}\;\mathrm{static}\;\mathrm{BCs}\\ \; & \end{array}$$

(14c) $$\begin{array}{cc} \; & r\left(x\right)=\int_{0}^{L}\phi \left(x-t,L_{c}\right)kv\left(t\right)dt\;\mathrm{Nonlocal}\;\mathrm{DD}\;\mathrm{elastic}\;\mathrm{foundation} \end{array}$$

A noteworthy result shows that the nonlocal integral Equation (14c) can be replaced with an equivalent differential problem and foundation boundary conditions according to the next proposition proved in Appendix B, starting from the results provided in [29].

**Proposition** **1** (Equivalence property for a displacement-driven (DD) elastic foundation). *The reactions r of the integral equation Equation (14c) with the special kernel Equation (3) provides a unique solution of the constitutive differential equation of elastic foundation:*

(15) $$r\left(x\right)-L_{c}^{2}\;\partial_{x}^{2}r\left(x\right)=kv\left(x\right),$$

*with $x\in \left[0,L\right]$, subject to the two homogeneous foundation boundary conditions (FBCs):*

(16) $$\left\{\begin{array}{c} {\left.\partial_{x}r\left(x\right)\right|}_{x=0}-\frac{1}{L_{c}}r\left(0\right)=0\\ {\left.\partial_{x}r\left(x\right)\right|}_{x=L}+\frac{1}{L_{c}}r\left(L\right)=0. \end{array}\right.$$

Accordingly, the DD convolution law Equation (14c) can be replaced with the differential equation in Equation (15) and the FBCs in Equation (16).

Hence, to solve the elastostatic model of a beam on a nonlocal DD model of the elastic foundation, reported in Box 1, we substitute the transverse displacement *v*, obtained from Equation (15), into Equation (14a). The elastostatic structural nonlocal differential problem is thus reported in Box 2.

Box 2 **Elastostatic structural differential problem of a beam on nonlocal DD elastic foundation.**

(17a) $$\begin{array}{cc} \; & \frac{I_{E}}{k}\partial_{x}^{4}r\left(x\right)-\frac{I_{E}L_{c}^{2}}{k}\partial_{x}^{6}r\left(x\right)+r\left(x\right)=q_{y}\left(x\right)\;\mathrm{Beam}\;\mathrm{elastic}\;\mathrm{equilibrium}\\ \; & \end{array}$$

(17b) $$\begin{array}{cc} \; & \left.\begin{array}{c} \left\{\frac{1}{k}r\left(x\right)-\frac{L_{c}^{2}}{k}\;\partial_{x}^{2}r\left(x\right),\right.\\ \frac{1}{k}\partial_{x}r\left(x\right)-\frac{L_{c}^{2}}{k}\;\partial_{x}^{3}r\left(x\right),\\ \frac{I_{E}}{k}\partial_{x}^{2}r\left(x\right)-\frac{I_{E}L_{c}^{2}}{k}\;\partial_{x}^{4}r\left(x\right),\\ {\left.-\frac{I_{E}}{k}\partial_{x}^{3}r\left(x\right)+\frac{I_{E}L_{c}^{2}}{k}\;\partial_{x}^{5}r\left(x\right)\right\}}_{x=\left\{0,L\right\}} \end{array}\right\}\;\mathrm{Kinematic}\;\mathrm{and}\;\mathrm{static}\;\mathrm{BCs}\\ \; & \end{array}$$

(17c) $$\begin{array}{cc} \; & \left.\begin{array}{c} {\left.\partial_{x}r\left(x\right)\right|}_{x=0}-\frac{1}{L_{c}}r\left(0\right)=0\;\;\;\;\;\\ {\left.\partial_{x}r\left(x\right)\right|}_{x=L}+\frac{1}{L_{c}}r\left(L\right)=0. \end{array}\right\}\;\mathrm{FBCs} \end{array}$$

The sixth-order differential equation of the elastic problem Equation (17a) can be solved by using four classical kinematic and static boundary conditions following from Equation (17b) in terms of reactions *r* and two FBCs Equation (17c).

The transverse displacement *v* in the beam interval $\left[0,L\right]$ is obtained by Equation (15) in terms of reactions *r*

(18) $$v\left(x\right)=\frac{1}{k}r\left(x\right)-\frac{L_{c}^{2}}{k}\;\partial_{x}^{2}r\left(x\right).$$

Further, bending moment and shear force fields of the beam are given by

(19) $$\begin{array}{c} M\left(x\right)=\frac{I_{E}}{k}\partial_{x}^{2}r\left(x\right)-\frac{I_{E}L_{c}^{2}}{k}\;\partial_{x}^{4}r\left(x\right)\\ T\left(x\right)=-\frac{I_{E}}{k}\partial_{x}^{3}r\left(x\right)+\frac{I_{E}L_{c}^{2}}{k}\;\partial_{x}^{5}r\left(x\right). \end{array}$$

It is worth noting that the elastostatic structural problem of a beam on nonlocal DD elastic foundation is solved without postulating the existence of any fictitious reactive force at the beam end points as it must be done in the modified Wieghardt model.

### Transverse Displacement of the Nonlocal Elastic Foundation Outside the Beam Interval

If the elastic foundation extends outside the beam interval $\left[0,L\right]$, we can evaluate the transverse displacement fields of the surface of the elastic foundation $v_{1DD}$, for x≤0, and $v_{2DD}$, for x≥L, by considering the following nonlocal expressions obtained by the RD model Equation (2)

(20) $$\left\{\begin{array}{cc} v_{1DD}\left(x\right)=\int_{0}^{L}\frac{1}{2L_{c}k}\exp\left(\frac{x-t}{L_{c}}\right)r\left(t\right)dt+C_{1}\exp\left(\frac{x}{L_{c}}\right) & \;\mathrm{for}\;x\leq 0\\ v_{2DD}\left(x\right)=\int_{0}^{L}\frac{1}{2L_{c}k}\exp\left(-\frac{x-t}{L_{c}}\right)r\left(t\right)dt+C_{2}\exp\left(-\frac{x-L}{L_{c}}\right) & \;\mathrm{for}\;x\geq L \end{array}\right.$$

where the reaction field *r* is the solution of the elastostatic structural problem of the beam on the nonlocal DD elastic foundation. The parameters $C_{1}$ and $C_{2}$ are introduced in order to fulfil the continuity requirement of the displacement field at the beam end points x=0 and x=L.

Since the continuity of the displacement field at x=0 and x=L requires

(21) $$v_{1DD}\left(0\right)=v\left(0\right),\;v_{2DD}\left(L\right)=v\left(L\right),$$

using Equation (20), the two parameters $C_{1}$ and $C_{2}$ are given in the following form:

(22) $$\left\{\begin{array}{c} C_{1}=v\left(0\right)-\int_{0}^{L}\frac{1}{2L_{c}k}\exp\left(-\frac{t}{L_{c}}\right)r\left(t\right)dt\\ C_{2}=v\left(L\right)-\int_{0}^{L}\frac{1}{2L_{c}k}\exp\left(-\frac{L-t}{L_{c}}\right)r\left(t\right)dt. \end{array}\right.$$

Hence, the transverse displacement fields Equation (20) of the surface of the elastic foundation $v_{1DD}$ and $v_{2DD}$ are

(23) $$\left\{\begin{array}{cc} v_{1DD}\left(x\right)=v\left(0\right)\exp\left(-\frac{\left|x\right|}{L_{c}}\right) & \;\mathrm{for}\;x\leq 0\\ v_{2DD}\left(x\right)=v\left(L\right)\exp\left(-\frac{x-L}{L_{c}}\right) & \;\mathrm{for}\;x\geq L. \end{array}\right.$$

**Remark** **1.** *The transverse displacement fields $v_{1DD}$ and $v_{2DD}$ can also be obtained by differentiating Equation (20) to obtain*

(24) $$\left\{\begin{array}{cc} \partial_{x}v_{1DD}\left(x\right)=\frac{1}{L_{c}}v_{1DD}\left(x\right) & \;\mathrm{for}\;x\leq 0\\ \partial_{x}v_{2DD}\left(x\right)=-\frac{1}{L_{c}}v_{2DD}\left(x\right)\; & \;\mathrm{for}\;x\geq L. \end{array}\right.$$

*The solution of the differential Equation (24) provides the transverse displacement fields of the surface of the elastic foundation in terms of two integration constants ${\hat{C}}_{1}$ and ${\hat{C}}_{2}$:*

(25) $$\left\{\begin{array}{cc} v_{1DD}\left(x\right)={\hat{C}}_{1}\exp\left(-\frac{\left|x\right|}{L_{c}}\right) & \;\mathrm{for}\;x\leq 0\\ v_{2DD}\left(x\right)={\hat{C}}_{2}\exp\left(-\frac{x}{L_{c}}\right)\; & \;\mathrm{for}\;x\geq L. \end{array}\right.$$

*Enforcing continuity of the displacement field at x=0 and x=L Equation (21), it results in*

(26) $${\hat{C}}_{1}=v\left(0\right),\;{\hat{C}}_{2}=v\left(L\right)\exp\left(\frac{L}{L_{c}}\right)$$

*so that the transverse displacement fields of the surface of the elastic foundation $v_{1DD}$ and $v_{2DD}$ are provided by inserting Equation (26) into Equation (25). The transverse displacement fields Equation (23) are thus recovered.*

**Remark** **2.** *The transverse displacement fields of the surface of the elastic foundation pertaining to the MRD nonlocal model $v_{1M}$, for x≤0, and $v_{2M}$, for x≥L, are given in the following form [25]:*

(27) $$\left\{\begin{array}{cc} \begin{array}{c} v_{1M}\left(x\right)=\int_{0}^{L}\frac{1}{2L_{c}k}\exp\left(\frac{x-t}{L_{c}}\right)r\left(t\right)dt\\ \;+\frac{A_{1}}{2L_{c}k}\exp\left(\frac{x}{L_{c}}\right)+\frac{A_{2}}{2L_{c}k}\exp\left(\frac{x-L}{L_{c}}\right) \end{array}\; & \;\mathrm{for}\;x\leq 0\\ \begin{array}{c} v_{2M}\left(x\right)=\int_{0}^{L}\frac{1}{2L_{c}k}\exp\left(-\frac{x-t}{L_{c}}\right)r\left(t\right)dt\\ \;+\frac{A_{1}}{2L_{c}k}\exp\left(-\frac{x}{L_{c}}\right)+\frac{A_{2}}{2L_{c}k}\exp\left(-\frac{x-L}{L_{c}}\right). \end{array}\; & \;\mathrm{for}\;x\geq L \end{array}\right.$$

*The two fictitious forces $A_{1}$ and $A_{2}$ are obtained by enforcing continuity of the displacement field at x=0 and x=L*

(28) $$v_{1M}\left(0\right)=v_{M}\left(0\right),\;v_{2M}\left(L\right)=v_{M}\left(L\right),$$

*with $v_{M}$ being the transverse displacement field of the surface of the elastic foundation obtained by the MRD nonlocal model. Substituting such forces in Equation (27), the transverse displacement fields $v_{1M}$ and $v_{2M}$ are given by*

(29) $$\left\{\begin{array}{cc} v_{1M}\left(x\right)=v_{M}\left(0\right)\exp\left(-\frac{\left|x\right|}{L_{c}}\right) & \;\mathrm{for}\;x\leq 0\\ v_{2M}\left(x\right)=v_{M}\left(L\right)\exp\left(-\frac{x-L}{L_{c}}\right) & \;\mathrm{for}\;x\geq L. \end{array}\right.$$

*It is worth noting that Equation (29) turn out to be coincident to the ones reported in [25] and to Equation (23) by replacing $v\left(0\right)$ and $v\left(L\right)$ with $v_{M}\left(0\right)$ and $v_{M}\left(L\right)$.*

## 5. Numerical Applications

We provide some numerical results of technical interest to illustrate the effectiveness of the proposed methodology for the analysis of Bernoulli–Euler beams on a nonlocal foundation. The free-beam (FF) under uniform load and simply supported beam (SS) under uniform load are considered. The solution of the elastostatic problem for a beam on a nonlocal DD elastic foundation is obtained by the nonlocal differential problem reported in Box 2 that has been solved using Mathematica software by Stephen Wolfram [38]. This solution is then compared with the MRD nonlocal model [25].

We consider the nonlocal elastostatic problem in a non-dimensional form by introducing the following non-dimensional quantities: abscissa ξ, nonnegative length-scale parameter λ, transverse displacement $v^{*}$, transverse load $q_{y}^{*}$, transverse force $F^{*}$, Winkler modulus $k^{*}$, reaction $r^{*}$, bending moment $M^{*}$, and shear force $T^{*}$

(30) $$\begin{array}{c} \xi =\frac{x}{L},\;\lambda =\frac{L_{c}}{L},\;v^{*}=\frac{v}{L},\;q_{y}^{*}=\frac{q_{y}L^{3}}{I_{E}},\;F^{*}=\frac{FL^{2}}{I_{E}}\\ k^{*}=\frac{kL^{4}}{I_{E}},\;r^{*}=\frac{rL^{3}}{I_{E}},\;M^{*}=\frac{ML}{I_{E}},\;T^{*}=\frac{TL^{2}}{I_{E}}. \end{array}$$

The non-dimensional length-scale parameter is $\lambda \in \left\{0^{+},0.10,0.20,0.30,0.40,0.50\right\}$, where $\lambda =0^{+}$ stands for λ→0, and the non-dimensional Winkler modulus is $k^{*}\in \left\{0,0.4,2,10,20\right\}$.

### 5.1. Free Beam on a Nonlocal Foundation Subject to a Uniformly Distributed Load

Let us consider an FF beam on a nonlocal elastic foundation subject to a non-dimensional uniform transverse load $q_{y}^{*}=-1$.

The solution of the beam on a nonlocal DD elastic foundation can be provided by solving Equation (17a) of Box 2 rewritten in the non-dimensional form

(31) $$-\partial_{\xi }^{6}r^{*}\left(\xi \right)+\frac{1}{\lambda^{2}}\partial_{\xi }^{4}r^{*}\left(\xi \right)+\frac{k^{*}}{\lambda^{2}}r^{*}\left(\xi \right)=-\frac{k^{*}}{\lambda^{2}}$$

equipped with the classical non-dimensional static boundary conditions at the beam end points following from Equation (17b), i.e., $M^{*}\left(0\right)=T^{*}\left(0\right)=M^{*}\left(1\right)=T^{*}\left(1\right)=0$, and the FBCs in Equation (17c) in the form

(32) $$\left\{\begin{array}{c} {\left.\partial_{\xi }^{2}r^{*}\left(\xi \right)\right|}_{\xi =0}-{\left.\lambda^{2}\partial_{\xi }^{4}r^{*}\left(\xi \right)\right|}_{\xi =0}=0\\ {\left.-\partial_{\xi }^{3}r^{*}\left(\xi \right)\right|}_{\xi =0}+{\left.\lambda^{2}\;\partial_{\xi }^{5}r^{*}\left(\xi \right)\right|}_{\xi =0}=0\\ {\left.\partial_{\xi }^{2}r^{*}\left(\xi \right)\right|}_{\xi =1}-{\left.\lambda^{2}\;\partial_{\xi }^{4}r^{*}\left(\xi \right)\right|}_{\xi =1}=0\\ {\left.-\partial_{\xi }^{3}r^{*}\left(\xi \right)\right|}_{\xi =1}+{\left.\lambda^{2}\;\partial_{\xi }^{5}r^{*}\left(\xi \right)\right|}_{\xi =1}=0\\ {\left.\partial_{\xi }r^{*}\left(\xi \right)\right|}_{\xi =0}-\frac{1}{\lambda }r^{*}\left(0\right)=0\\ {\left.\partial_{\xi }r^{*}\left(\xi \right)\right|}_{\xi =1}+\frac{1}{\lambda }r^{*}\left(1\right)=0. \end{array}\right.$$

The non-dimensional transverse displacement $v^{*}$ of the beam is then given by Equation (18) in terms of the non-dimensional foundation reactions $r^{*}$

(33) $$v^{*}\left(x\right)=\frac{1}{k^{*}}r^{*}\left(\xi \right)-\frac{\lambda^{2}}{k^{*}}\;\partial_{\xi }^{2}r^{*}\left(\xi \right).$$

The non-dimensional bending moment and shear force follow from Equation (19):

(34) $$\left\{\begin{array}{c} M^{*}\left(\xi \right)=\frac{1}{k^{*}}\partial_{\xi }^{2}r^{*}\left(\xi \right)-\frac{\lambda^{2}}{k^{*}}\;\partial_{\xi }^{4}r^{*}\left(\xi \right)\\ T^{*}\left(\xi \right)=-\frac{1}{k^{*}}\partial_{\xi }^{3}r^{*}\left(\xi \right)+\frac{\lambda^{2}}{k^{*}}\;\partial_{\xi }^{5}r^{*}\left(\xi \right). \end{array}\right.$$

The non-dimensional transverse displacement of the surface of the elastic foundation outside the beam interval $\left[0,L\right]$ can be obtained by Equation (23) in the following form:

(35) $$\left\{\begin{array}{cc} v_{1DD}^{*}\left(\xi \right)=v^{*}\left(0\right)\exp\left(-\frac{\left|\xi \right|}{\lambda }\right) & \;\mathrm{for}\;\xi \leq 0\\ v_{2DD}^{*}\left(\xi \right)=v^{*}\left(1\right)\exp\left(-\frac{\xi -1}{\lambda }\right) & \;\mathrm{for}\;\xi \geq 1, \end{array}\right.$$

where $v^{*}\left(0\right)$ and $v^{*}\left(1\right)$ are the non-dimensional displacements at the beam end points.

The solution of the FF beam on the nonlocal DD elastic foundation yields the classical solution of the FF beam on a Winkler foundation by letting $\lambda \to 0^{+}$.

The non-dimensional transverse deflection $v^{*}$, reaction $r^{*}$, and bending moment $M^{*}$ at the midpoint ξ=1/2 of the free beam subjected to a uniform transverse load are presented in Table 1, Table 2 and Table 3 using the DD and MRD nonlocal models for several values of the non-dimensional Winkler parameter $k^{*}$ and length-scale parameter λ.

**Figure 2:**
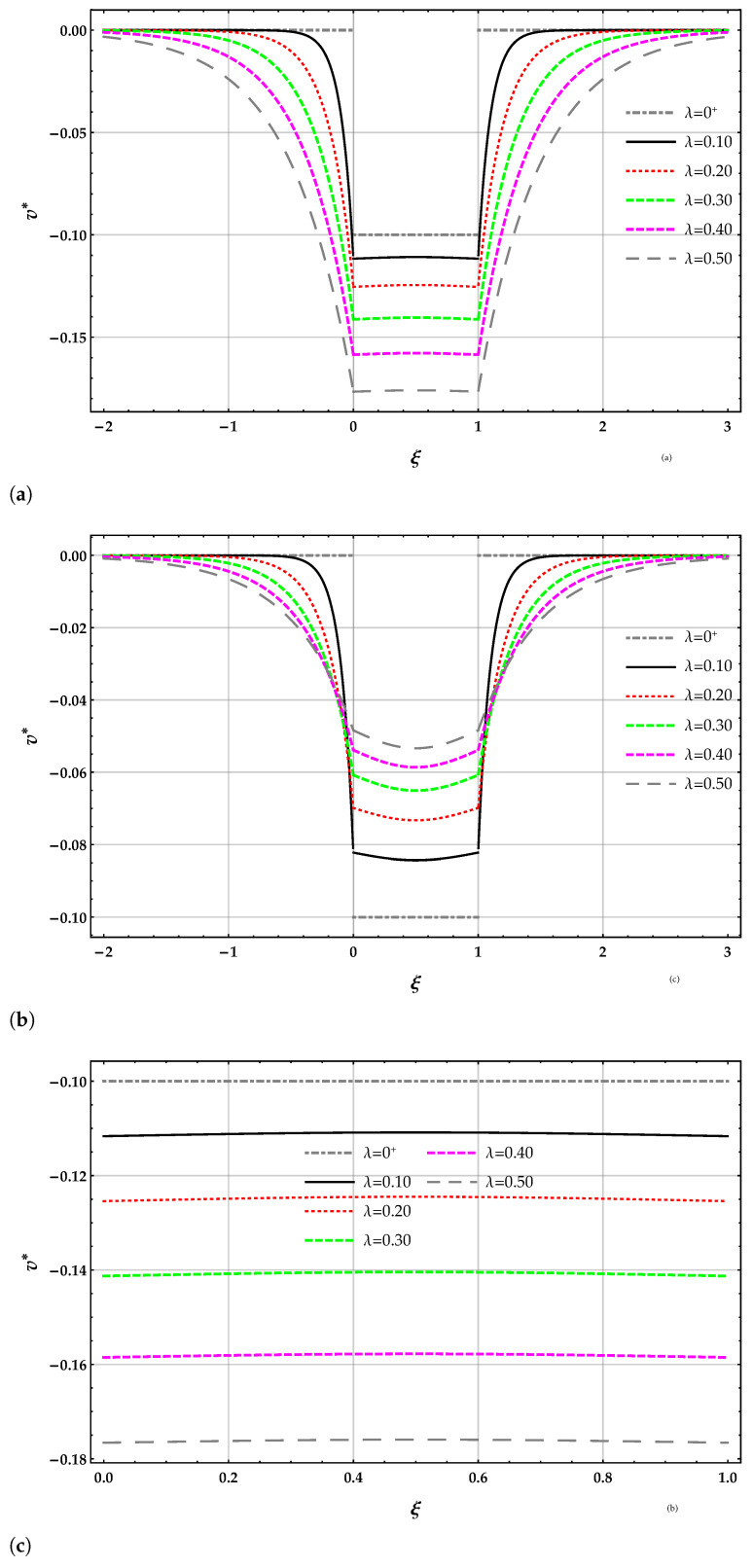

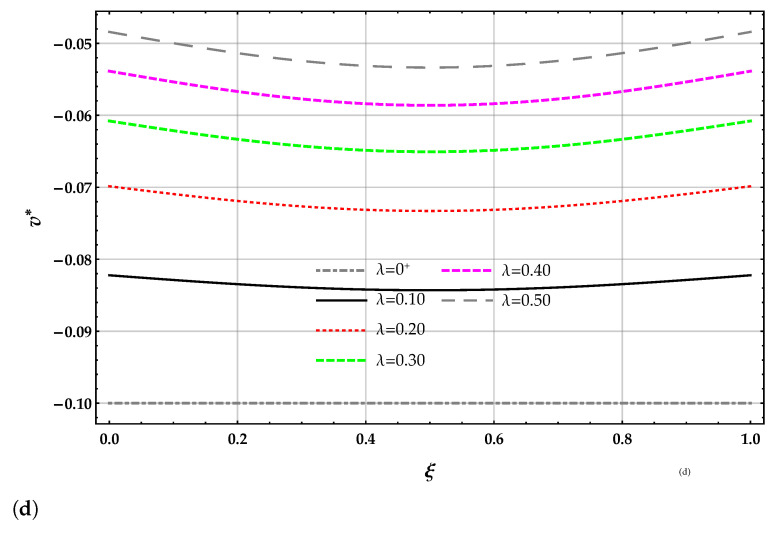
FF−q beam. Plots of the non-dimensional transverse displacement $v^{*}$ of the surface of the elastic foundation for increasing values of the non-dimensional non-local parameter λ in the set $\left\{0^{+},0.1,0.2,0.3,0.4,0.5\right\}$ with $k^{*}=10$: (**a**) DD method, (**b**) MRD method, (**c**) zoom of the beam deflection using the DD method, (**d**) zoom of the beam deflection using the MRD method.

The two non-dimensional fictitious forces $A_{1}^{*}=A_{2}^{*}$ of the beam on the nonlocal MRD elastic foundation are reported in Table 4. The non-dimensional transverse displacement $v^{*}$ of the surface of the nonlocal elastic foundation in the interval $\left[0.5,3\right]$ is reported in Table 5 for the DD and MRD nonlocal models for increasing values of Winkler parameter $k^{*}$ and for the length-scale parameter λ=0.5. The non-dimensional transverse displacement $v^{*}$ of the surface of the elastic foundation in the interval $\left[-2,3\right]$, obtained by the DD and MRD methods, are reported in Figure 2a,c, respectively, in terms of the length-scale parameter λ with $k^{*}=10$. The zoom of the beam deflection is reported in Figure 2b for the DD method and in Figure 2d for the MRD method.

Note that the displacements $v^{*}\left(1/2\right)$ of the surface of the foundation obtained by solving the beam on the nonlocal DD model of the elastic foundation are greater than the corresponding ones provided by the MRD model for a given λ and $k^{*}$; see Table 1 and Table 5. The displacements of the surface of the foundation obtained by solving the beam on the nonlocal DD and MRD models of the elastic foundation decrease for increasing values of the Winkler parameter $k^{*}$ at a given value of λ. The displacement $v^{*}\left(1/2\right)$ obtained by solving the beam on the nonlocal DD elastic foundation increases for increasing values of λ at a given value of the Winkler parameter $k^{*}$ and decreases for the nonlocal MRD model.

**Figure 3:**
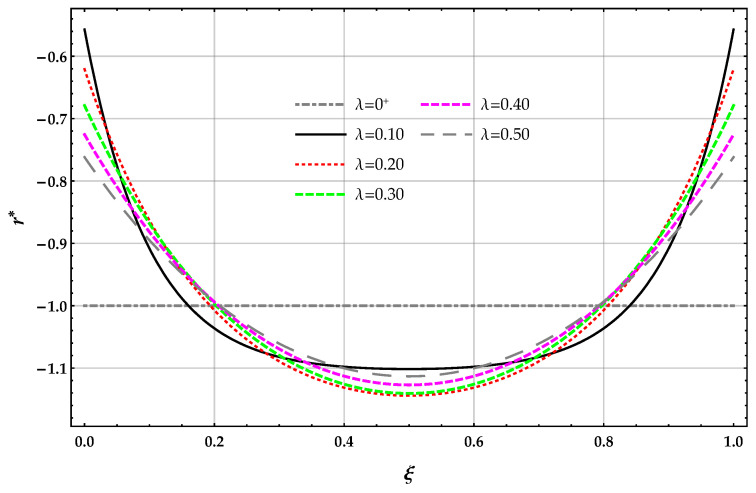
FF−q beam. DD method: plots of the non-dimensional reaction $r^{*}$ of the elastic foundation for increasing values of the non-dimensional non-local parameter λ in the set $\left\{0^{+},0.1,0.2,0.3,0.4,0.5\right\}$ with $k^{*}=10$.

The non-dimensional reaction $r^{*}$ applied on the surface of the elastic foundation and obtained by the DD model is plotted in Figure 3 in terms of the length-scale parameter λ with $k^{*}=10$.

The reactions $r^{*}\left(1/2\right)$ obtained by solving the beam on the nonlocal DD model of the elastic foundation are greater than the corresponding ones provided by the MRD method for a given λ and $k^{*}$; see Table 2. The reactions obtained by solving the beam on the nonlocal DD model of the elastic foundation decrease for increasing values of the Winkler parameter $k^{*}$ at a given value of λ and increases for the nonlocal MRD method. The reactions $r^{*}\left(1/2\right)$ obtained by solving the beam on the nonlocal DD elastic foundation increases for increasing values of λ at a given value of the Winkler parameter $k^{*}$ and then decreases. The reactions $r^{*}\left(1/2\right)$ obtained by solving the beam on the nonlocal MRD model of the elastic foundation decreases for increasing values of λ at a given value of the Winkler parameter $k^{*}$.

**Figure 4:**
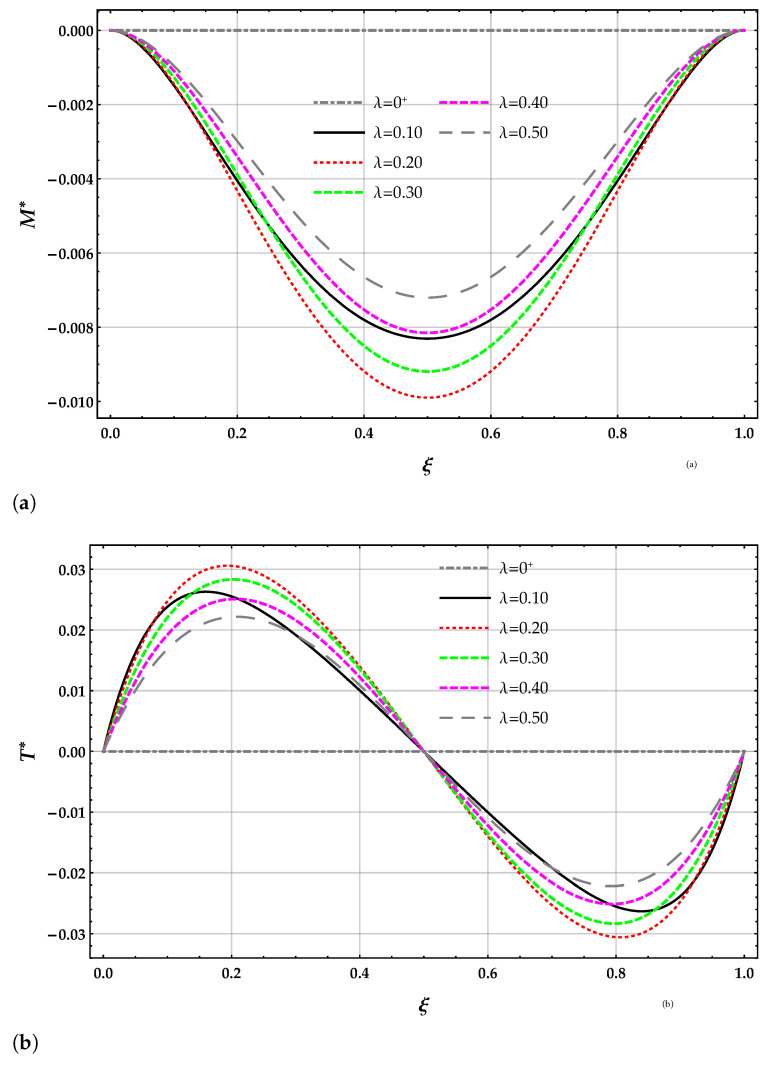
FF−q beam. Plots for increasing values of the non-dimensional non-local parameter λ in the set $\left\{0^{+},0.1,0.2,0.3,0.4,0.5\right\}$ with $k^{*}=10$ of (**a**) non-dimensional bending moment $M^{*}$ using the DD method and (**b**) non-dimensional shear force $T^{*}$ using the DD method.

The non-dimensional bending moment $M^{*}$ and shear force $T^{*}$ obtained by the DD model are plotted in terms of the length-scale parameter λ with $k^{*}=10$ in Figure 4a,b. The slope of the bending moment and, hence, the shear force $T^{*}$ at the beam end points of the free beam vanishes for any value of the length-scale parameter λ.

The bending moment $M^{*}$ obtained by solving the beam on the nonlocal DD model of the elastic foundation is smaller than the corresponding ones provided by the MRD model for a given λ and $k^{*}$; see Table 3. The bending moment $M^{*}$ obtained by solving the beam on the nonlocal DD and MRD models of the elastic foundation decreases for increasing values of the Winkler parameter $k^{*}$ at a given value of λ. The bending moment $M^{*}$ obtained by solving the beam on the nonlocal DD elastic foundation increases for increasing values of λ at a given value of the Winkler parameter $k^{*}$ and then decreases. The bending moment $M^{*}$ obtained by solving the beam on the nonlocal MRD model of the elastic foundation increases for increasing values of λ at a given value of the Winkler parameter $k^{*}$.

**Figure 5:**
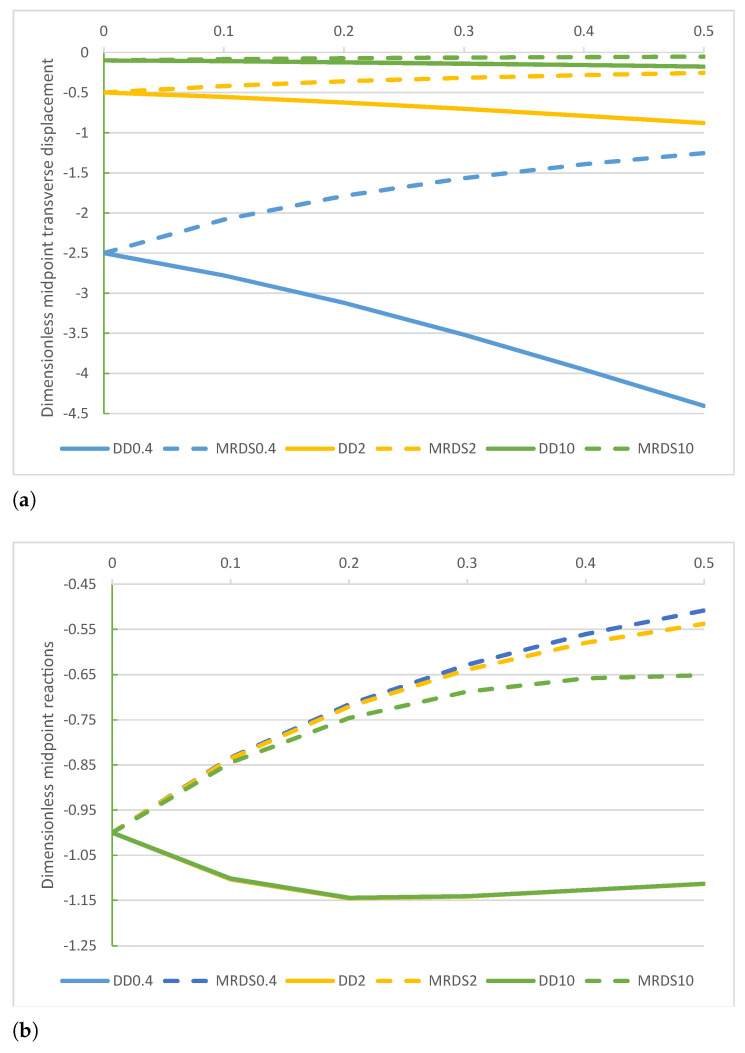

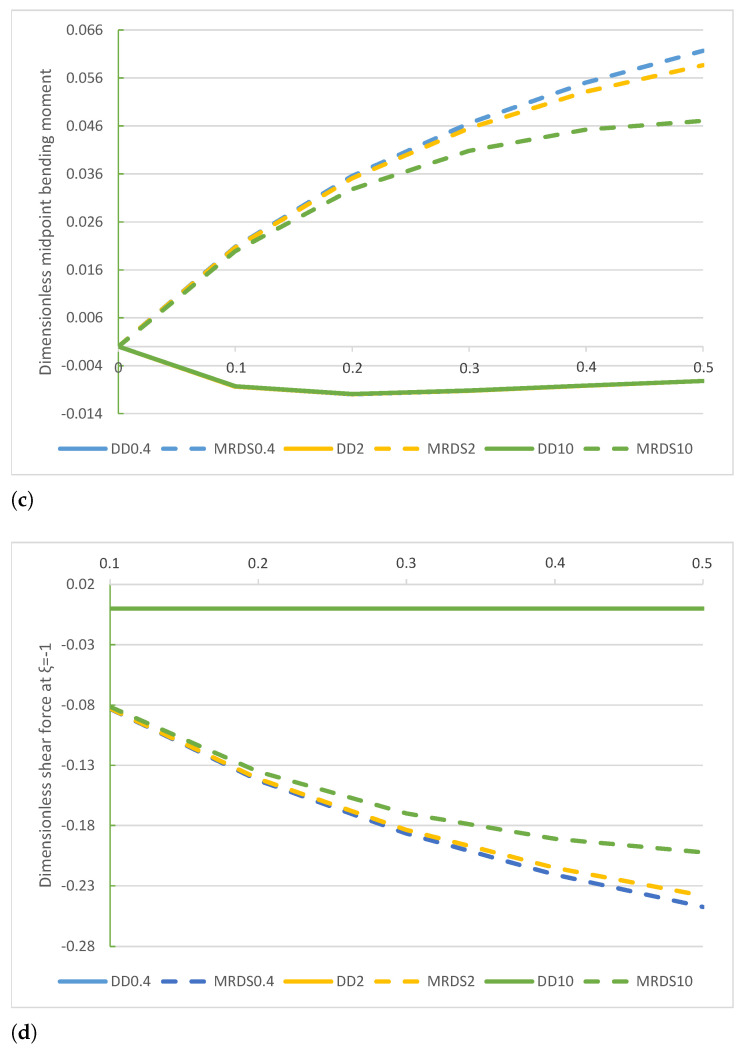
FF−q beam. Comparison of the DD and MRD methods for increasing values of the non-dimensional non-local parameter λ in the set $\left\{0^{+},0.1,0.2,0.3,0.4,0.5\right\}$ with $k^{*}=\left\{0.4,2,10\right\}$: (**a**) non-dimensional transverse midpoint displacement $v^{*}\left(1/2\right)$, (**b**) dimensionless reaction $r^{*}\left(1/2\right)$, (**c**) non-dimensional bending moment $M^{*}\left(1/2\right)$, and (**d**) non-dimensional shear force $T^{*}\left(-1\right)$.

A comparison of the non-dimensional displacement $v^{*}\left(1/2\right)$, reaction $r^{*}\left(1/2\right)$, bending moment $M^{*}\left(1/2\right)$, and shear force $T^{*}\left(-1\right)$ obtained by the DD and MRD models are plotted in Figure 5a–d.

### 5.2. Simply Supported Beam on a Nonlocal Foundation Subject to a Uniformly Distributed Load

Let us consider a SS beam on a nonlocal elastic foundation subject to a non-dimensional uniform transverse load $q_{y}^{*}=-1$.

The solution of the beam on a nonlocal DD elastic foundation using the differential approach can be provided by solving Equation (17a) of Box 2 rewritten in the non-dimensional form

(36) $$-\partial_{\xi }^{6}r^{*}\left(\xi \right)+\frac{1}{\lambda^{2}}\partial_{\xi }^{4}r^{*}\left(\xi \right)+\frac{k^{*}}{\lambda^{2}}r^{*}\left(\xi \right)=-\frac{k^{*}}{\lambda^{2}}$$

equipped with the classical kinematic and static boundary conditions at the beam end points following from Equation (17b), i.e., $v^{*}\left(0\right)=M^{*}\left(0\right)=v^{*}\left(1\right)=M^{*}\left(1\right)=0$, and the FBCs following from Equation (17c) in the non-dimensional form

(37) $$\left\{\begin{array}{c} r^{*}\left(0\right)-{\left.\lambda^{2}\partial_{\xi }^{2}r^{*}\left(\xi \right)\right|}_{\xi =0}=0\\ {\left.\partial_{\xi }^{2}r^{*}\left(\xi \right)\right|}_{\xi =0}-{\left.\lambda^{2}\;\partial_{\xi }^{4}r^{*}\left(\xi \right)\right|}_{\xi =0}=0\\ r^{*}\left(1\right)-{\left.\lambda^{2}\partial_{\xi }^{2}r^{*}\left(\xi \right)\right|}_{\xi =1}=0\\ {\left.\partial_{\xi }^{2}r^{*}\left(\xi \right)\right|}_{\xi =1}-{\left.\lambda^{2}\;\partial_{\xi }^{4}r^{*}\left(\xi \right)\right|}_{\xi =1}=0\\ {\left.\partial_{\xi }r^{*}\left(\xi \right)\right|}_{\xi =0}-\frac{1}{\lambda }r^{*}\left(0\right)=0\\ {\left.\partial_{\xi }r^{*}\left(\xi \right)\right|}_{\xi =1}+\frac{1}{\lambda }r^{*}\left(1\right)=0. \end{array}\right.$$

The non-dimensional transverse displacement $v^{*}$ of the beam is then given by Equation (18) in terms of the non-dimensional foundation reactions $r^{*}$

(38) $$v^{*}\left(x\right)=\frac{1}{k^{*}}r^{*}\left(\xi \right)-\frac{\lambda^{2}}{k^{*}}\;\partial_{\xi }^{2}r^{*}\left(\xi \right).$$

The non-dimensional bending moment $M^{*}$ and shear force $T^{*}$ follow from Equation (19).

The non-dimensional classical displacement and bending moment at the midpoint of the SS beam with no elastic foundation (NEF) are $-\frac{5}{384}=-0.0130208$ and $\frac{1}{8}=0.125$, respectively, and the non-dimensional shear force at ξ=1 is $\frac{1}{2}=0.5$.

The DD and MRD models yield the classical solution of a beam on a Winkler foundation by letting $\lambda \to 0^{+}$.

The non-dimensional transverse displacement $v^{*}$, foundation reactions $r^{*}$, and bending moment $M^{*}$ at the midpoint ξ=1/2 and the shear force $T^{*}\left(1\right)$ of the SS beam are presented in Table 6, Table 7, Table 8 and Table 9 using the DD and MRD models for several values of non-dimensional Winkler parameter $k^{*}$ and non-dimensional length-scale parameter λ. The two non-dimensional fictitious forces $A_{1}^{*}=A_{2}^{*}$ of the beam on the nonlocal MRD elastic foundation are reported in Table 10.

The displacements $v^{*}\left(1/2\right)$ of the surface of the foundation obtained by the DD model are greater than the corresponding ones provided by the MRD model for a given λ and $k^{*}$.

**Figure 6:**
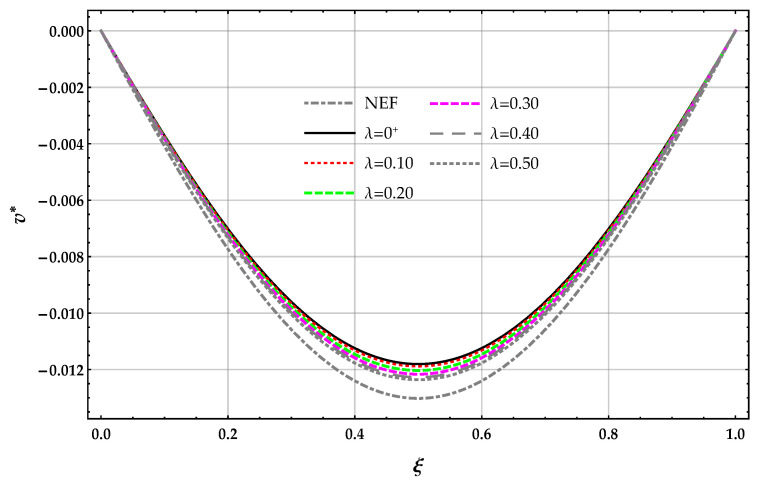
SS−q beam. DD method: plots of the non-dimensional transverse displacement $v^{*}$ of the surface of the elastic foundation for increasing values of the non-dimensional non-local parameter λ in the set $\left\{0^{+},0.1,0.2,0.3,0.4,0.5\right\}$ with $k^{*}=10$.

The non-dimensional transverse displacement $v^{*}$ obtained by the DD model is reported in Figure 6 in terms of the length-scale parameter λ with $k^{*}=10$.

The non-dimensional transverse displacement $v^{*}\left(1/2\right)$ obtained by solving the beam on the nonlocal DD elastic foundation are greater than the corresponding ones provided by the MRD model for a given λ and $k^{*}$; see Table 6. The displacement $v^{*}\left(1/2\right)$ obtained by solving the beam on the nonlocal DD and MRD models of the elastic foundation decreases for increasing values of the Winkler parameter $k^{*}$ at a given value of λ.

The displacement $v^{*}\left(1/2\right)$ obtained by solving the beam on the nonlocal DD model of the elastic foundation increases for increasing values of λ at a given valued of the Winkler parameter $k^{*}$ and decreases for the nonlocal MRD model.

**Figure 7:**
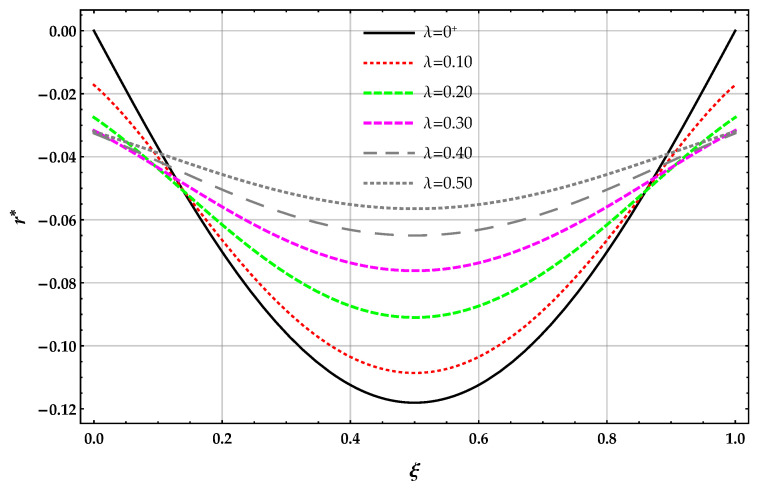
SS−q beam. DD method: plots of the non-dimensional reaction $r^{*}$ of the elastic foundation for increasing values of the non-dimensional non-local parameter λ in the set $\left\{0^{+},0.1,0.2,0.3,0.4,0.5\right\}$ with $k^{*}=10$.

The non-dimensional reaction $r^{*}$ applied on the surface of the elastic foundation and obtained by the DD model is plotted in Figure 7 in terms of the length-scale parameter λ with $k^{*}=10$.

The reactions $r^{*}\left(1/2\right)$ obtained by solving the beam on the nonlocal DD model of the elastic foundation are smaller than the corresponding ones provided by the MRD model for a given λ and $k^{*}$; see Table 7. The reactions $r^{*}\left(1/2\right)$ obtained by solving the beam on the nonlocal DD and MRD models decrease for increasing values of the Winkler parameter $k^{*}$ at a given value of λ and then increases. The reactions $r^{*}\left(1/2\right)$ obtained by solving the beam on the nonlocal DD model of the elastic foundation decreases for increasing values of λ at a given value of the Winkler parameter $k^{*}$. On the contrary, the reactions $r^{*}\left(1/2\right)$ obtained by solving the beam on the nonlocal MRD model of the elastic foundation increases for increasing values of λ at a given value of the Winkler parameter $k^{*}$.

**Figure 8:**
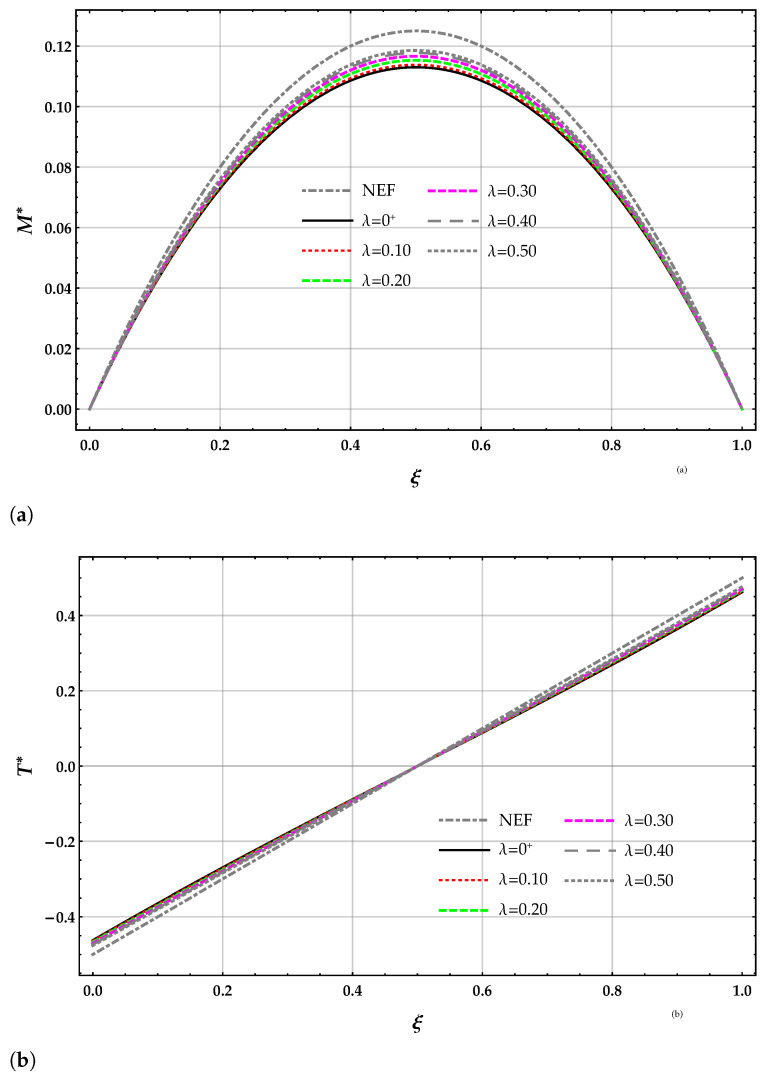
SS−q beam. Plots for increasing values of the non-dimensional non-local parameter λ in the set $\left\{0^{+},0.1,0.2,0.3,0.4,0.5\right\}$ with $k^{*}=10$ of (**a**) non-dimensional bending moment $M^{*}$ using the DD method and (**b**) non-dimensional shear force $T^{*}$ using the DD method.

The non-dimensional bending moment $M^{*}$ and shear force $T^{*}$ of the DD model are plotted in terms of the length-scale parameter λ with $k^{*}=10$ in Figure 8a,b. The non-dimensional bending moment $M^{*}$ and shear force $T^{*}$ depend on the value the length-scale parameter λ.

The bending moment $M^{*}\left(1/2\right)$ obtained by solving the beam on the nonlocal DD elastic foundation are greater than the corresponding ones provided by the MRD model for a given λ and $k^{*}$; see Table 8. The bending moment $M^{*}\left(1/2\right)$ obtained by solving the beam on the nonlocal DD and MRD models of the elastic foundation decrease for increasing values of the Winkler parameter $k^{*}$ at a given value of λ. The bending moment $M^{*}\left(1/2\right)$ obtained by solving the beam on the nonlocal DD elastic foundation increases for increasing values of λ at a given value of the Winkler parameter $k^{*}$. The bending moment $M^{*}\left(1/2\right)$ obtained by solving the beam on the nonlocal MRD model of the elastic foundation decreases for increasing values of λ at a given value of the Winkler parameter $k^{*}$.

**Figure 9:**
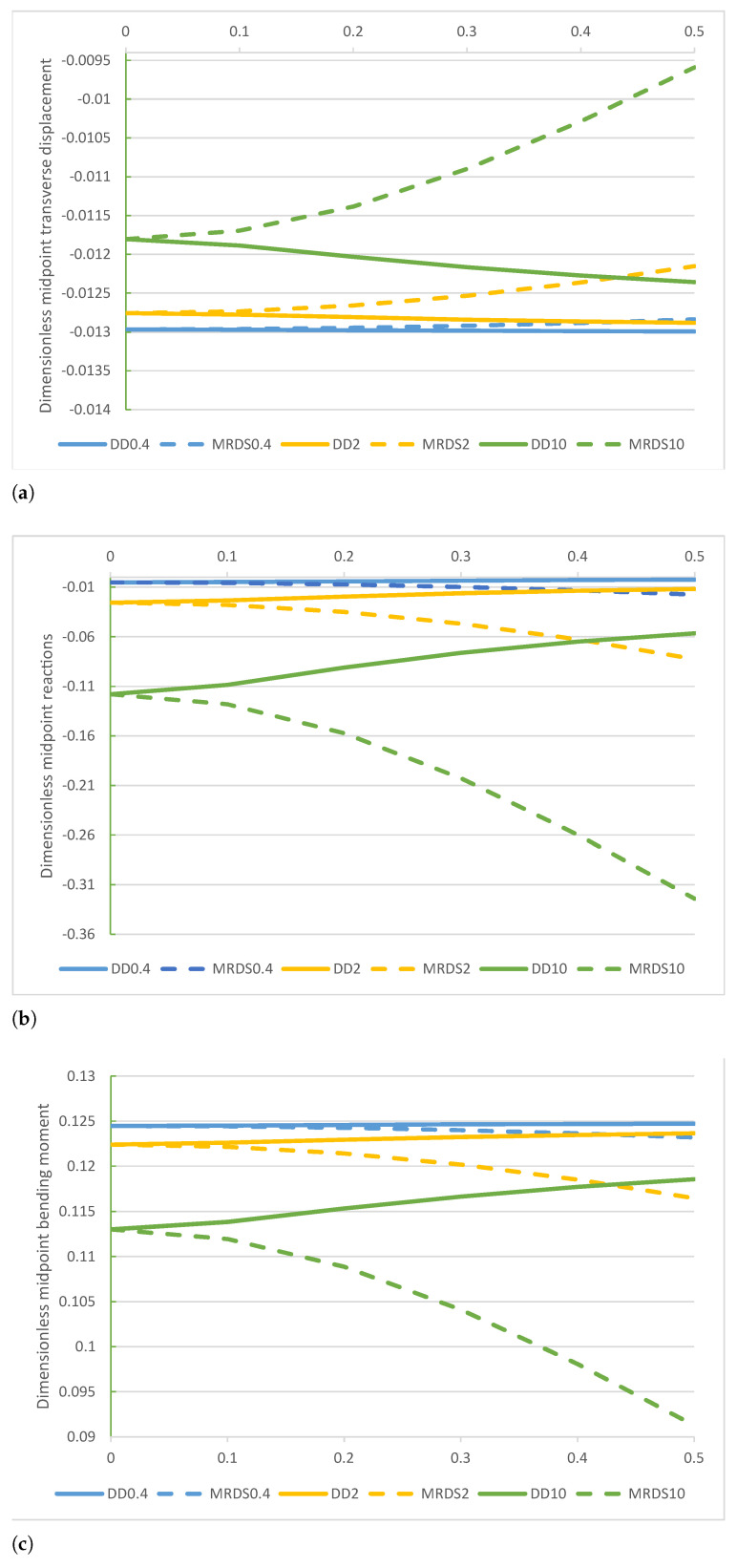

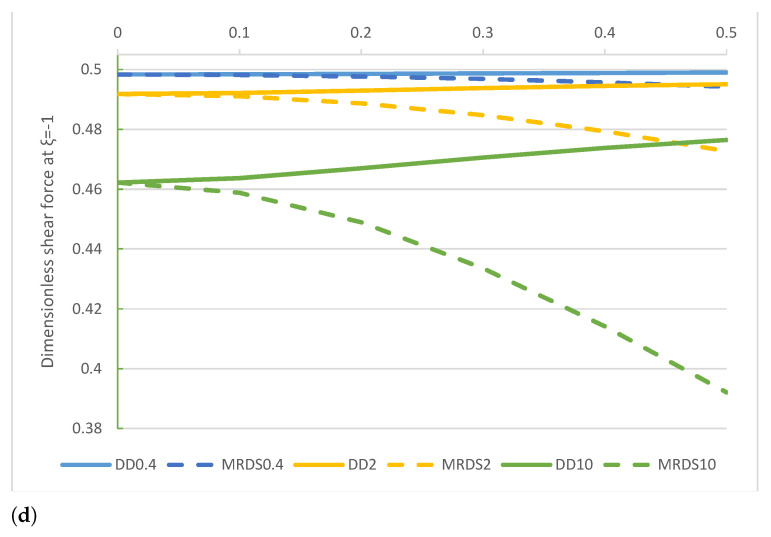
SS−q beam. Comparison of the DD and MRD methods for increasing values of the non-dimensional non-local parameter λ in the set $\left\{0^{+},0.1,0.2,0.3,0.4,0.5\right\}$ with $k^{*}=\left\{0.4,2,10\right\}$: (**a**) non-dimensional transverse midpoint displacement $v^{*}\left(1/2\right)$, (**b**) dimensionless reaction $r^{*}\left(1/2\right)$, (**c**) non-dimensional bending moment $M^{*}\left(1/2\right)$, and (**d**) non-dimensional shear force $T^{*}\left(1\right)$.

A comparison of the midpoint non-dimensional displacement $v^{*}\left(1/2\right)$, reaction $r^{*}\left(1/2\right)$, bending moment $M^{*}\left(1/2\right)$, and shear force T(1) of the DD and MRD methods are plotted in Figure 9a–d. Numerical outcomes of non-dimensional shear force T(1) of DD and MRD methods are given in Table 9 while numerical results of non-dimensional parameters $A_{1}^{*}=A_{2}^{*}$ obtained by the MRD method are provided in Table 10.

## 6. Concluding Remarks

The bending behavior of a beam resting on an elastic foundation was investigated by using a well-posed displacement-driven (DD) nonlocal integral approach. The nonlocal equations governing the relevant structural problem were formulated by conveniently replacing the DD convolution integral characterizing the soil elasticity model with an equivalent nonlocal differential relation and constitutive boundary conditions. No concentrated forces at beam boundary (x=0, x=L) are needed in the proposed DD formulation to obtain well-posedness of the relevant structural elastostatic problem. This is a significant advancement with respect to the original Wieghardt nonlocal foundation model [4] and its modification by Van Langendonck and Sollazzo [24,25], where additional constitutive terms were imposed to obtain well-posedness but without a clear physical interpretation. the effects of various factors, such as the Winkler modulus and nonlocal length-scale parameter, on bending elastic responses of free and simply supported beams under different loading conditions and external constraints of applicative interest were established, examined, and discussed. Extensive numerical data were given in tabular form for several values of geometric and constitutive non-dimensional parameters. These data could provide useful benchmarks for future studies. The proposed DD foundation model were shown to provide a significant assessment of nonlocal effects for any loading system and kinematic boundary conditions. Difficulties and issues involved in some classical analyses are thus overcome ab initio. Future developments will be focused on reasonable extensions of the DD nonlocal approach to be adopted for the simulation of ultra-elastic models of soils.

## Figures and Tables

**Table 1:** Free beam subjected to a non-dimensional uniform load $q_{y}^{*}=-1$. Non-dimensional maximum displacement $v^{*}\left(1/2\right)$ versus the non-dimensional length-scale parameter λ evaluated by the non-dimensional Winkler parameter $k^{*}\in \left\{0.4,2,10,20\right\}$ in the DD and MRD models.

v${}^{*}$ (1/2)									
λ	**DD**					**MRD**			
	k${}^{*}$ = 0.4	k${}^{*}$ = 2	k${}^{*}$ = 10	k${}^{*}$ = 20		k${}^{*}$ = 0.4	k${}^{*}$ = 2	k${}^{*}$ = 10	k${}^{*}$ = 20
$0^{+}$	−2.5	−0.5	−0.1	−0.05		−2.5	−0.5	−0.1	−0.05
0.1	−2.7775	−0.555285	−0.110845	−0.0552931		−2.08434	−0.41767	−0.0843029	−0.0425966
0.2	−3.11944	−0.623643	−0.124485	−0.0620923		−1.78773	−0.359129	−0.0732901	−0.0374405
0.3	−3.51736	−0.703245	−0.140422	−0.0700698		−1.56541	−0.315348	−0.0650625	−0.0335266
0.4	−3.95023	−0.789841	−0.157764	−0.0787552		−1.39259	−0.28135	−0.0586113	−0.030365
0.5	−4.40376	−0.880569	−0.175931	−0.0878521		−1.25437	−0.254164	−0.0533617	−0.0277079

**Table 2:** Free beam subjected to a non-dimensional uniform load $q_{y}^{*}=-1$. Non-dimensional midpoint foundation reaction $r^{*}\left(1/2\right)$ versus the non-dimensional length-scale parameter λ evaluated by the non-dimensional Winkler parameter $k^{*}\in \left\{0.4,2,10,20\right\}$ in the DD and MRD models.

r${}^{*}$ (1/2)									
λ	**DD**					**MRD**			
	k${}^{*}$ = 0.4	k${}^{*}$ = 2	k${}^{*}$ = 10	k${}^{*}$ = 20		k${}^{*}$ = 0.4	k${}^{*}$ = 2	k${}^{*}$ = 10	k${}^{*}$ = 20
$0^{+}$	−1.0	−1.0	−1.0	−1.0		−1.0	−1.0	−1.0	−1.0
0.1	−1.10354	−1.10322	−1.10166	−1.09974		−0.833821	−0.835754	−0.845019	−0.85574
0.2	−1.14542	−1.14522	−1.14425	−1.14305		−0.71566	−0.721065	−0.746027	−0.773076
0.3	−1.14127	−1.14117	−1.14065	−1.14001		−0.627843	−0.638892	−0.687364	−0.735528
0.4	−1.12745	−1.12739	−1.1271	−1.12673		−0.560558	−0.579707	−0.658509	−0.729175
0.5	−1.11353	−1.1135	−1.11332	−1.1131		−0.507919	−0.537668	−0.651312	−0.742323

**Table 3:** Free beam subjected to a non-dimensional uniform load $q_{y}^{*}=-1$. Non-dimensional midpoint bending moment $M^{*}\left(1/2\right)$ versus the non-dimensional length-scale parameter λ evaluated by the non-dimensional Winkler parameter $k^{*}\in \left\{0.4,2,10,20\right\}$ in the Displacement-Driven (DD) and Modified Reaction-Driven (MRD) models.

M${}^{*}$ (1/2)									
λ	**DD**					**MRD**			
	k${}^{*}$ = 0.4	k${}^{*}$ = 2	k${}^{*}$ = 10	k${}^{*}$ = 20		k${}^{*}$ = 0.4	k${}^{*}$ = 2	k${}^{*}$ = 10	k${}^{*}$ = 20
$0^{+}$	0	0	0	0		0	0	0	0
0.1	−0.00840319	−0.00838687	−0.00830616	−0.00820727		0.0207943	0.0206397	0.0198977	0.0190375
0.2	−0.00995599	−0.00994611	−0.00989699	−0.00983623		0.0355889	0.0350958	0.0328139	0.0303315
0.3	−0.00922294	−0.00921768	−0.00919144	−0.00915884		0.0465999	0.0455298	0.0408201	0.0361086
0.4	−0.00816841	−0.00816543	−0.00815058	−0.00813209		0.0550571	0.0531471	0.0452469	0.038086
0.5	−0.00721239	−0.00721058	−0.00720154	−0.00719027		0.0616985	0.0586827	0.0470779	0.037633

**Table 4:** Free beam subjected to a non-dimensional uniform load $q_{y}^{*}=-1$. Non-dimensional parameters $A_{1}^{*}=A_{2}^{*}$ versus the non-dimensional length-scale parameter λ evaluated by the non-dimensional Winkler parameter $k^{*}\in \left\{0.4,2,10,20\right\}$ in the MRD model.

$A_{1}^{*}=A_{2}^{*}$				
λ	**MRD**			
	k${}^{*}$ = 0.4	k${}^{*}$ = 2	k${}^{*}$ = 10	k${}^{*}$ = 20
0.1	−0.0832594	−0.0829661	−0.0815572	−0.0799202
0.2	−0.142532	−0.141251	−0.135315	−0.128836
0.3	−0.186708	−0.183624	−0.170018	−0.156337
0.4	−0.220721	−0.214963	−0.191062	−0.169231
0.5	−0.247531	−0.23823	−0.202257	−0.172651

**Table 5:** Free beam subjected to a non-dimensional uniform load $q_{y}^{*}=-1$ with the non-dimensional length-scale parameter λ=0.5. Non-dimensional displacement $v^{*}\left(\xi \right)$ evaluated by the non-dimensional Winkler parameter $k^{*}\in \left\{0.4,2,10,20\right\}$ in the DD and MRD models.

$v^{*}\left(\xi \right)$ with λ=0.5									
ξ	**DD**					**MRD**			
	k${}^{*}$ = 0.4	k${}^{*}$ = 2	k${}^{*}$ = 10	k${}^{*}$ = 20		k${}^{*}$ = 0.4	k${}^{*}$ = 2	k${}^{*}$ = 10	k${}^{*}$ = 20
0.5	−4.40376	−0.880569	−0.175931	−0.0878521		−1.25437	−0.254164	−0.0533617	−0.0277079
0.6	−4.40379	−0.880604	−0.175967	−0.0878876		−1.25407	−0.253872	−0.0531278	−0.0275208
0.7	−4.40389	−0.880706	−0.176068	−0.0879886		−1.25317	−0.253021	−0.0524435	−0.0269726
0.8	−4.40405	−0.880858	−0.17622	−0.0881401		−1.25176	−0.251679	−0.0513622	−0.0261034
0.9	−4.40423	−0.88104	−0.176402	−0.0883215		−1.24996	−0.249964	−0.049975	−0.0249838
1.0	−4.40442	−0.881232	−0.176594	−0.0885133		−1.24794	−0.24804	−0.0484141	−0.0237196
1.2	−2.95237	−0.590707	−0.118374	−0.0593323		−0.836521	−0.166266	−0.032453	−0.0158997
1.4	−1.97903	−0.395963	−0.0793487	−0.0397716		−0.560737	−0.111451	−0.021753	−0.0106579
1.6	−1.32659	−0.265422	−0.053189	−0.0266597		−0.375873	−0.0747081	−0.0145821	−0.0071442
1.8	−0.889237	−0.177918	−0.0356536	−0.0178705		−0.251955	−0.0500784	−0.00977464	−0.0047889
2.0	−0.596073	−0.119262	−0.0238994	−0.011979		−0.168891	−0.0335685	−0.00655214	−0.00321009
2.2	−0.39956	−0.0799436	−0.0160202	−0.00802975		−0.113211	−0.0225017	−0.0043920	−0.00215179
2.4	−0.267833	−0.0535878	−0.0107387	−0.0053825		−0.0758875	−0.0150833	−0.00294407	−0.00144239
2.6	−0.179534	−0.035921	−0.00719835	−0.003608		−0.0508689	−0.0101106	−0.00197347	−0.000966862
2.8	−0.120345	−0.0240785	−0.0048252	−0.00241851		−0.0340984	−0.00677737	−0.00132285	−0.00064810
3.0	−0.0806698	−0.0161403	−0.00323443	−0.00162118		−0.0228569	−0.00454301	−0.000886735	−0.000434439

**Table 6:** Simply supported beam subjected to a non-dimensional uniform load $q_{y}^{*}=-1$. Non-dimensional maximum displacement $v^{*}\left(1/2\right)$ versus the non-dimensional length-scale parameter λ evaluated by the non-dimensional Winkler parameter $k^{*}\in \left\{0.4,2,10,20\right\}$ in the DD and MRD models.

v${}^{*}$ (1/2)									
λ	**DD**					**MRD**			
	k${}^{*}$ = 0.4	k${}^{*}$ = 2	k${}^{*}$ = 10	k${}^{*}$ = 20		k${}^{*}$ = 0.4	k${}^{*}$ = 2	k${}^{*}$ = 10	k${}^{*}$ = 20
$0^{+}$	−0.0129674	−0.0127579	−0.011804	−0.0107944		−0.0129674	−0.0127579	−0.011804	−0.0107944
0.1	−0.0129713	−0.0127769	−0.0118858	−0.0109322		−0.0129621	−0.0127325	−0.0116961	−0.0106152
0.2	−0.0129781	−0.0128101	−0.012031	−0.0111806		−0.0129464	−0.012657	−0.0113839	−0.0101115
0.3	−0.0129842	−0.0128399	−0.0121636	−0.0114121		−0.0129203	−0.0125331	−0.010899	−0.00937019
0.4	−0.0129891	−0.0128637	−0.0122715	−0.0116035		−0.0128839	−0.0123637	−0.0102855	−0.00849743
0.5	−0.0129929	−0.0128826	−0.0123577	−0.0117587		−0.0128374	−0.0121524	−0.00959096	−0.00758798

**Table 7:** Simply supported beam subjected to a non-dimensional uniform load $q_{y}^{*}=-1$. Non-dimensional midpoint foundation reaction $r^{*}\left(1/2\right)$ versus the non-dimensional length-scale parameter λ evaluated by the non-dimensional Winkler parameter $k^{*}\in \left\{0.4,2,10,20\right\}$ in the DD and MRD models.

r${}^{*}$ (1/2)									
λ	**DD**					**MRD**			
	k${}^{*}$ = 0.4	k${}^{*}$ = 2	k${}^{*}$ = 10	k${}^{*}$ = 20		k${}^{*}$ = 0.4	k${}^{*}$ = 2	k${}^{*}$ = 10	k${}^{*}$ = 20
$0^{+}$	−0.00518695	−0.0255157	−0.11804	−0.215888		−0.00518695	−0.0255157	−0.11804	−0.215888
0.1	−0.00473987	−0.0233446	−0.108595	−0.199791		−0.00568254	−0.0279081	−0.128154	−0.232559
0.2	−0.00392519	−0.0193724	−0.0909833	−0.169134		−0.00716682	−0.0350271	−0.157386	−0.279296
0.3	−0.00325168	−0.016078	−0.0761649	−0.142938		−0.00963245	−0.0467014	−0.202683	−0.347708
0.4	−0.00275059	−0.0136205	−0.0649725	−0.122886		−0.0130672	−0.0626567	−0.259779	−0.427556
0.5	−0.00237493	−0.011774	−0.0564754	−0.107486		−0.0174544	−0.082531	−0.324063	−0.509754

**Table 8:** Simply supported beam subjected to a non-dimensional uniform load $q_{y}^{*}=-1$. Non-dimensional midpoint bending moment $M^{*}\left(1/2\right)$ versus the non-dimensional length-scale parameter λ evaluated by the non-dimensional Winkler parameter $k^{*}\in \left\{0.4,2,10,20\right\}$ in the DD and MRD models.

M${}^{*}$ (1/2)									
λ	**DD**					**MRD**			
	k${}^{*}$ = 0.4	k${}^{*}$ = 2	k${}^{*}$ = 10	k${}^{*}$ = 20		k${}^{*}$ = 0.4	k${}^{*}$ = 2	k${}^{*}$ = 10	k${}^{*}$ = 20
$0^{+}$	0.124473	0.122406	0.112995	0.103036		0.124473	0.122406	0.112995	0.103036
0.1	0.124512	0.122598	0.113826	0.104439		0.124421	0.122156	0.111935	0.101277
0.2	0.124582	0.122936	0.115305	0.106976		0.124266	0.121414	0.108867	0.0963317
0.3	0.124643	0.123235	0.116638	0.109306		0.124009	0.120195	0.104103	0.0890578
0.4	0.124691	0.123472	0.11771	0.111212		0.123651	0.118529	0.0980778	0.0805024
0.5	0.124729	0.123658	0.118562	0.112747		0.123194	0.116452	0.0912613	0.0715989

**Table 9:** Simply supported beam subjected to a non-dimensional uniform load $q_{y}^{*}=-1$. Non-dimensional shear force $T^{*}\left(1\right)$ versus the non-dimensional length-scale parameter λ evaluated by the non-dimensional Winkler parameter $k^{*}\in \left\{0.4,2,10,20\right\}$ in the DD and MRD models.

T${}^{*}$ (1)									
λ	**DD**					**MRD**			
	k${}^{*}$ = 0.4	k${}^{*}$ = 2	k${}^{*}$ = 10	k${}^{*}$ = 20		k${}^{*}$ = 0.4	k${}^{*}$ = 2	k${}^{*}$ = 10	k${}^{*}$ = 20
$0^{+}$	0.49834	0.491834	0.462207	0.430842		0.49834	0.491834	0.462207	0.430842
0.1	0.498415	0.492192	0.463669	0.433139		0.498175	0.491035	0.4588	0.425162
0.2	0.498576	0.492972	0.466991	0.43863		0.49768	0.488657	0.448937	0.409178
0.3	0.498743	0.493787	0.470565	0.444756		0.496858	0.484753	0.433596	0.385589
0.4	0.498889	0.494496	0.473746	0.450343		0.495712	0.479413	0.414148	0.357704
0.5	0.499009	0.495087	0.476435	0.45515		0.494248	0.472753	0.392079	0.328478

**Table 10:** Simply supported beam subjected to a non-dimensional uniform load $q_{y}^{*}=-1$. Non-dimensional parameters $A_{1}^{*}=A_{2}^{*}$ versus the non-dimensional length-scale parameter λ evaluated by the non-dimensional Winkler parameter $k^{*}\in \left\{0.4,2,10,20\right\}$ in the MRD model.

$A_{1}^{*}=A_{2}^{*}$				
λ	**MRD**			
	k${}^{*}$ = 0.4	k${}^{*}$ = 2	k${}^{*}$ = 10	k${}^{*}$ = 20
0.1	0.000165929	0.00081521	0.00375032	0.00682117
0.2	0.000662923	0.00324183	0.0146081	0.0260151
0.3	0.00148862	0.00722391	0.0314937	0.0543281
0.4	0.0026391	0.0126718	0.0528961	0.087776
0.5	0.00410896	0.0194672	0.0771786	0.1228

## Data Availability

The data presented in this study are available within this article. Further inquiries may be directed to the authors.

## List of Acronyms

DD	Displacement-Driven
FBCs	Foundation Boundary Conditions
FF	Free-Free beam
MFBCs	Modified Foundation Boundary Conditions
MRD	Modified Reaction-Driven
NEF	No Elastic Foundation
SS	Simply Supported beam
RD	Reaction-Driven
RDFBCs	Reaction-Driven foundation boundary conditions

## Appendix A

The Modified Reaction-Driven (MRD) nonlocal model of a Wieghardt elastic foundation postulates the existence of two fictitious forces at the end points x=0 and x=L of the beam. Denoting by $A_{1}$ and $A_{2}$ such fictitious forces, the modified Wieghardt convolution integral Equation (6) is rewritten hereafter for convenience:

(A1) $$v\left(x\right)=\int_{0}^{L}\phi \left(x-t,L_{c}\right)\frac{r\left(t\right)}{k}dt+\frac{A_{1}}{2L_{c}k}\exp\left(-\frac{x}{L_{c}}\right)+\frac{A_{2}}{2L_{c}k}\exp\left(\frac{x-L}{L_{c}}\right).$$

The nonlocal integral equation Equation (A1) can be replaced with an equivalent differential formulation and modified foundation boundary conditions (MFBCs) according to the next proposition, which can be proven following a similar reasoning to the one reported in Appendix B.

**Proposition** **A1** (Equivalence property for the MRD model of a Wieghardt foundation.). *The transversal displacement v obtained from the MRD Equation (A1) with the special kernel Equation (3) provides a unique solution of the differential equation*

(A2) $$\frac{1}{L_{c}^{2}}v\left(x\right)-\partial_{x}^{2}v\left(x\right)=\frac{1}{kL_{c}^{2}}r\left(x\right),$$

*with $x\in \left[0,L\right]$, subject to the two homogeneous modified foundation boundary conditions (MFBCs):*

(A3) $$\left\{\begin{array}{c} {\left.\partial_{x}v\left(x\right)\right|}_{x=0}-\frac{1}{L_{c}}v\left(0\right)+\frac{A_{1}}{L_{c}^{2}k}=0\\ {\left.\partial_{x}v\left(x\right)\right|}_{x=L}+\frac{1}{L_{c}}v\left(L\right)-\frac{A_{2}}{L_{c}^{2}k}=0. \end{array}\right.$$

The elastostatic structural problem of a beam on a nonlocal MRD foundation can be obtained by substituting the reaction *r*, obtained from Equation (A2), into Equation (11). Hence, we obtain the differential governing equation of a beam on a MRD model of the Wieghardt foundation in terms of the transverse displacement *v*

(A4) $$I_{E}\partial_{x}^{4}v\left(x\right)-kL_{c}^{2}\partial_{x}^{2}v\left(x\right)+kv\left(x\right)=q_{y}\left(x\right)$$

equipped with the classical kinematic and static boundary conditions by specifying $\left\{v,\partial_{x}v,M,\partial_{x}M\right\}$ at the beam end points $x=\left\{0,L\right\}$ and the MFBCs in Equation (A3).

It is apparent that the four integration constants following from Equation (A4) and the two unknown fictitious forces $A_{1}$ and $A_{2}$ at the end points of the Wieghardt foundation can be obtained by solving the linear sistem of equations obtained by imposing the four classical (local) constraint conditions of the beam and the two MFBCs in Equation (A3).

The foundation reactions *r* follow from Equation (A2) or equivalently from Equation (11) in terms of the transverse displacement *v* as

(A5) $$r\left(x\right)=kv\left(x\right)-kL_{c}^{2}\partial_{x}^{2}v\left(x\right),$$

where the bending moment is $M\left(x\right)=I_{E}\partial_{x}^{2}v\left(x\right)$ and the shear force is $T\left(x\right)=-I_{E}\partial_{x}^{3}v\left(x\right)$.

## Appendix B

Let us prove the following result.

**Proposition** **A2** (Equivalence property for the displacement-driven (DD) model of an elastic foundation). *The following nonlocal constitutive law equipped with the bi-exponential kernel Equation (3)*

(A6) $$r\left(x\right)=\int_{0}^{L}\phi \left(x-t,L_{c}\right)kv\left(t\right)dt.$$

*with $x\in \left[0,L\right]$ is equivalent to the differential relation*

(A7) $$r\left(x\right)-L_{c}^{2}\;\partial_{x}^{2}r\left(x\right)=kv\left(x\right)$$

*subject to the following two foundation boundary conditions (FBC)*

(A8) $$\left\{\begin{array}{c} {\left.\partial_{x}r\left(x\right)\right|}_{x=0}-\frac{1}{L_{c}}r\left(0\right)=0\\ {\left.\partial_{x}r\left(x\right)\right|}_{x=L}+\frac{1}{L_{c}}r\left(L\right)=0. \end{array}\right.$$

**Proof.** Since the bi-exponential averaging function is given by

(A9) $$\phi \left(x-t,L_{c}\right)=\frac{1}{2L_{c}}\exp\left(-\frac{\left|x-t\right|}{L_{c}}\right),$$

and the integral convolution Equation (A6) can be rewritten in the form

(A10) $$r\left(x\right)=\int_{0}^{x}\phi \left(x-t,L_{c}\right)kv\left(t\right)dt+\int_{x}^{L}\phi \left(x-t,L_{c}\right)kv\left(t\right)dt,$$

a direct evaluation provides the first derivative of the foundation reactions *r*:

(A11) $$\begin{array}{c} \partial_{x}r\left(x\right)=\frac{k}{2L_{c}}v\left(x\right)-\frac{1}{L_{c}}\int_{0}^{x}\phi \left(x-t,L_{c}\right)\;kv\left(t\right)dt+\\ \;\;\;-\frac{k}{2L_{c}}v\left(x\right)+\frac{1}{L_{c}}\int_{x}^{L}\phi \left(x-t,L_{c}\right)\;kv\left(t\right)dt\\ \;\;=-\frac{1}{L_{c}}\int_{0}^{x}\phi \left(x-t,L_{c}\right)\;kv\left(t\right)dt\\ \;\;\;\;+\frac{1}{L_{c}}\int_{x}^{L}\phi \left(x-t,L_{c}\right)\;kv\left(t\right)dt. \end{array}$$

The second derivative of the convolution Equation (A10) follows from Equation (A11) to obtain

(A12) $$\begin{array}{c} \partial_{x}^{2}r\left(x\right)=-\frac{k}{2L_{c}^{2}}v\left(x\right)+\frac{1}{L_{c}^{2}}\int_{0}^{x}\phi \left(x-t,L_{c}\right)\;kv\left(t\right)dt+\\ \;\;\;-\frac{k}{2L_{c}^{2}}v\left(x\right)+\frac{1}{L_{c}^{2}}\int_{x}^{L}\phi \left(x-t,L_{c}\right)\;kv\left(t\right)dt\\ \;\;=-\frac{k}{L_{c}^{2}}v\left(x\right)+\frac{1}{L_{c}^{2}}\int_{0}^{L}\phi \left(x-t,L_{c}\right)\;kv\left(t\right)dt. \end{array}$$

Recalling Equation (A6) and rearranging the terms in Equation (A12), Equation (A7) is recovered. The FBCs in Equation (A8) of the nonlocal model follow by evaluating Equation (A11) at the beam boundary points x=0 and x=L. In fact, we have at x=0

(A13) $${\left.\partial_{x}r\left(x\right)\right|}_{x=0}=\frac{1}{L_{c}}\left[\int_{0}^{L}\phi \left(-\xi ,L_{c}\right)\;kv\left(t\right)dt\right]=\frac{1}{L_{c}}r\left(0\right)$$

and the FBC in Equation (A8)${}_{1}$ is recovered. Analogously, setting x=L in Equation (A11), we obtain

(A14) $${\left.\partial_{x}r\left(x\right)\right|}_{x=L}=-\frac{1}{L_{c}}\left[\int_{0}^{L}\phi \left(L-t,L_{c}\right)\;kv\left(t\right)dt\right]=-\frac{1}{L_{c}}r\left(L\right)$$

and the FBC in Equation (A8)${}_{2}$ is recovered. The uniqueness of the solution of Equation (A7) is consequent to the fact that the homogeneous differential problem (with v(x)=0), subject to the FBCs, admits only the trivial solution. □
